# Design of Enterprise Financial Information Fusion Sharing System Based on Blockchain Technology

**DOI:** 10.1155/2022/5402444

**Published:** 2022-09-08

**Authors:** Xiang Xue

**Affiliations:** Jiangsu Vocational College of Finance and Economics, Jiangsu, Huaian 223003, China

## Abstract

In the process of global economic integration, the rapid development of various industries not only brings various opportunities but also presents new challenges to enterprises. With the continuous expansion of enterprise scale, more and more enterprises join the trend of international competition, and the internal system of enterprise group organization is complex, resulting in the weakening of the management and control ability of the enterprise itself and the decline of operating efficiency. In order to improve this situation, the fusion and sharing of financial information has become a necessary development strategy to optimize management, strengthen internal control systems, and improve business performance in the era of big data. Technology promotes the development of productivity, and with the continuous maturity of computer technology, blockchain technology comes into being. Blockchain technology is of great significance in promoting industrial transformation and technological innovation. The development of the new era should strengthen the basic research of blockchain and regard blockchain as an important breakthrough for independent innovation of core technologies. This work first analyzes the necessity and feasibility of applying blockchain technology to the enterprise financial information fusion and sharing system. Then, it specifically describes how the blockchain technology is applied to the enterprise financial information fusion and sharing system, and improves the enterprise financial information fusion and sharing system through the blockchain technology. This forms an enterprise financial information fusion and sharing system between companies, which improves the limitations and disadvantages of the current financial sharing platform. Finally, using the AHP to analyze and compare the indicators of the traditional financial sharing model and the financial information fusion sharing model under the blockchain technology, it is concluded that the blockchain technology brings positive changes to the enterprise financial information fusion sharing model.

## 1. Introduction

Since the beginning of the 21st century, with the development of productivity driven by science and technology and the emergence of industrial transformation, new requirements and tests have been put forward for financial management. The world's scientific and technological ecological landscape and corporate economic structure are constantly being reshaped, and new-generation scientific and technological theories such as blockchain, artificial intelligence, and big data are no longer limited to the computer field. The continuous maturation of the new generation of technology has brought new vitality and vitality to traditional industries, promoted the transformation and upgrading of the industry, and changed the existing system model. As a world economic power, blockchain technology plays an important role in future economic development and industrial innovation. The earliest application of this technology is as the underlying technology of emerging digital currencies, such as Bitcoin and Litecoin, which are supported by such technologies. Its essence is a distributed accounting method based on cryptography, which is represented by a chain structure divided into six layers: data layer, network layer, consensus layer, incentive layer, contract layer, and application layer. Blockchain technology has four characteristics: immutability, uniqueness required to represent value, smart contracts, and decentralization [1–7].

The development of information technology has broken the barriers of the global economy, making the global economy more closely followed by a large number of mergers and acquisitions of enterprises and the development of globalization. Under this circumstance, it becomes more and more difficult for enterprises to manage the company's financial affairs. In addition, the differences in local policies lead to more and more confusions in the financial management of enterprises. The difficulty of management is obviously increased. Under this circumstance, in order to pursue an efficient financial management model, some foreign companies began to adopt the separation of financial functions within the enterprise and reengineer the financial process, and established an independent individual agency to engage in all financial works, and financial shared services came into being. With the development of financial shared services, various financial service software types have emerged, and enterprises often use a variety of software due to different business needs. The business model of enterprises is constantly innovating and developing. The requirements of modern society for accounting are not only limited to the field of financial accounting but also to the field of management. This transformation means that the problem of distortion of accounting data is not only limited to the internal accounting personnel of the enterprise. It is also the main content of the management personnel during the relevant work period. In the accounting industry, the problem of information fraud is a fundamental problem that should be paid attention to. In actual operation, because each department is an independent entity and each software database is not interoperable, the problem of information islands is difficult to deal with, and the efficiency is low, and the contradictions between departments are intensified. Most of the existing financial sharing construction schemes revolve around a single system. For example, the financial sharing construction of ZTE group takes the ERP system as the main core, but there is a lack of corresponding typical cases for reference in the multisystem integration construction [8–14].

However, with the continuous advancement of information technology, technologies such as blockchain have emerged. The relevant characteristics of blockchain technology and its technical characteristics can effectively communicate and integrate data, and distributed ledgers can meet the requirements of the accounting industry. It improves the accounting quality of enterprises, effectively solves the problems related to information islands, realizes software interoperability and re-division of power, and provides technical support for the multisystem integration of enterprises. Here, the financial sharing platform came into being, and major enterprises are eager to try, but the current financial sharing model still has problems such as imperfect organizational process and low data security. This provides a place for the blockchain to enter the enterprise financial sharing platform. The blockchain technology itself has unique advantages: decentralization, trustlessness, and a unique distributed ledger form. These advantages can be used precisely. To optimize the structure of the financial sharing platform, we promote the application, thereby improving the efficiency of financial work and expanding the economic benefits of enterprises. Therefore, blockchain technology is one of the rare ways to solve the problem of financial sharing construction of small- and medium-sized enterprises at this stage [15–20].

This work first analyzes the necessity and feasibility of applying blockchain technology to the enterprise financial information fusion and sharing system. Then, it specifically describes how the blockchain technology is applied to the enterprise financial information fusion and sharing system, and improves the enterprise financial information fusion and sharing system through the blockchain technology. This forms an enterprise financial information fusion and sharing system between companies, which improves the limitations and disadvantages of the current financial sharing platform. Finally, using the AHP to analyze and compare the indicators of the traditional financial sharing model and the financial information fusion sharing model under the blockchain technology, it is concluded that the blockchain technology brings positive changes to the enterprise financial information fusion sharing model.

## 2. Related Work

Literature [21] found that the adoption of accounting information software in nonlisted enterprises was positively correlated with the improvement of enterprise production efficiency. The more perfect the information software, the more obvious the effect, and the information software could improve the decision-making level by broadening the manager's data and information channels to provide more efficient support for the manager's management decision-making. Literature [22] believed that based on the financial shared service center, it could more scientifically and accurately analyze the problems existing in enterprise operation, and provide important support for enterprise budget management and capital management. Reference [23] explored how the financial sharing center could speed up the capital turnover rate, jointly build a reasonable capital management mechanism, and improve the service efficiency of the sharing center. The paper argued that the fund management model of the financial sharing center should change its thinking, build a new fund model, strengthen the prior control, and budget mechanism to reasonably plan the available funds in the region. Literature [24] believed that in the process of process reengineering in the process of enterprise construction, the process of sorting out the context would result in the re-division of enterprise power. This process was undoubtedly full of contradictions. Due to different management perspectives and thinking dimensions, the business side and the financial side would have different performance appraisal standards for the same target process. These different performance appraisal purposes would also bring difficulties to the construction of industry-finance integration. Literature [25] believed that the sharing center should pursue high quality and low risk based on the security of information capital system and customer-level service. Financial sharing construction should not simply be imitated, but should be constantly innovated according to the development of current productivity. Literature [26] found that the development of financial sharing in various industries is uneven through DEA efficiency research, and the efficiency value of enterprises established later was significantly higher than that of earlier enterprises. Early-stage companies had begun to enter a period of decline, so the current construction of shared services continued to innovate. Literature [27] believed that the construction of a financial sharing center should take the physical layer, the definition layer, the network layer, the collaboration layer, and the application layer as the main pillars. Literature [28] believed that financial sharing was a new management model; that is, the enterprise group separates some technical and some functional resources of its subsidiaries with lower organizational levels to achieve specialized centralized leadership. In addition, there was still a certain degree of competition among the leadership; that is to say, different subsidiaries and business units could realize the sharing of resources such as organization, technology, and personnel, which was the core idea of the financial sharing model. Literature [29] believed that by building a financial sharing center, the professionalism of financial work could be improved. And through the centralized processing and integration of information and data to improve the quality of accounting work, it could also make the ability of financial personnel more targeted, and provide business decision support for enterprises in a faster and more efficient way. Literature [30] believed that the effective implementation of financial shared services should be optimized and adjusted through performance management, because performance management could make the behavioral decisions of enterprise employees and managers restrained in an effective way. This enabled them to clearly recognize and fulfill their responsibilities, and has an incentive for everyone to adjust their status and achieve their job goals, so as to provide better services and gain relevant advantages in market competition.

Literature [31] believed that the Internet technology enables information to be transmitted and shared among various organizations in the enterprise, and the business data of each business link could also flow fully through the system according to the process. This provided favorable conditions for the management to keep abreast of material data information and capital use, so that the formulation and execution of business plans could be more accurate and traceable. Literature [32] believed that modern information technology could help enterprises build financial shared service centers. This was conducive to business sorting, forming standardized processes, improving automated processing procedures, and reducing manual errors and related costs. The reengineering of business processes had improved work efficiency and provided many favorable conditions for enterprise integration construction. Literature [33] proposed that the key elements of the construction of enterprise group financial shared service center were location selection, process reengineering, management assessment, and strategic planning by studying the enterprise financial sharing model. Although the development of the Internet had affected the enterprise management model, the organizational structure had changed and the business process had been re-integrated. However, Internet information technology also served as a favorable opportunity to promote the development of financial sharing, and promote enterprises to pay attention to and carry out in-depth integration of real estate and finance. Literature [34] believed that the scalability and modular structure of the private chain based on blockchain technology could meet the differentiated information access needs of enterprises, and meet the information needs of different levels and dimensions while ensuring real-time updates and transaction. Reference [35] studied the shared services based on blockchain technology, and it believed that making full use of the characteristics of blockchain could break the sense of distrust between people. This built a relational model of people, organizations, and technology that enabled business automation. It believed that blockchain solved the problem of trust between people, integrated modern database technology, cryptography, network management incentive mechanism, and other sciences and would lead the innovation of financial business and the logical basis of accounting in the future. Literature [36] believed that because blockchain technology had triggered disruptive innovation reforms on traditional economic transactions and payment settlement models, blockchain had become the most promising technology field for current applications and investment. The decentralization and trust mechanism of the blockchain would provide a natural development soil for the sharing economy and innovate the development model of the sharing economy. Reference [37] took the payment business as the entry point and believed that embedding the blockchain in the financial shared service center could simplify the review process and strictly control the business transaction and financial processing process. This could better ensure the accuracy and security of information, and the simplified process was flattened, which fundamentally improved the quality and efficiency of information transmission.

## 3. Methods

This work first analyzes the necessity and feasibility of applying blockchain technology to the enterprise financial information fusion and sharing system. Then, it specifically describes how the blockchain technology is applied to the enterprise financial information fusion and sharing system, and improves the enterprise financial information fusion and sharing system through the blockchain technology. This forms an enterprise financial information fusion and sharing system between companies, which improves the limitations and disadvantages of the current financial sharing platform. Finally, using the AHP to analyze and compare the indicators of the traditional financial sharing model and the financial information fusion sharing model under the blockchain technology, it is concluded that the blockchain technology brings positive changes to the enterprise financial information fusion sharing model.

### 3.1. Blockchain and Financial Sharing

A blockchain is a chained data structure that stores data into corresponding blocks in chronological order and connects these blocks. From its basic characteristics, it can also be understood as a distributed ledger protected by cryptography, which can effectively avoid the phenomenon of tampering with financial data. The data information is stored in the computer network through the blockchain and, at the same time, has the characteristics of decentralization and distribution. The information stored through the blockchain is not owned by group companies or individuals, but is open to everyone, ensuring the accuracy of data information. Blockchain technology is a distributed ledger, which is characterized by decentralization and a trust mechanism. On the blockchain, the work of storing, compiling data, and storing information can be performed, so the blockchain can also be regarded as a database in essence. Linkability is an obvious feature of the block, so the blockchain can also be regarded as a way to track products; that is, the blockchain system can be used to directly query the transaction information of each node. In addition, each node in the blockchain is equal, and there is no central node, so there is no phenomenon of using nodes to control or manipulate other nodes, so that the security of information and data is guaranteed to a certain extent.

Financial sharing is a new management model based on the continuous development of modern technology and informatization. Its key lies in the analysis and processing of the business process of financial personnel. Financial data, after unified integration, are aggregated to the internal financial sharing platform of the enterprise for processing, especially for work with large workload, high repetition, and easy scale standardization. For the purpose of improving efficiency, financial sharing aims to provide professional, efficient, and intelligent financial services to the company, and its ultimate purpose is to reduce the asymmetry of financial information, so as to achieve the goal of optimal allocation of financial information sources.

### 3.2. Feasibility of Blockchain Technology Application

**Figure 1:**
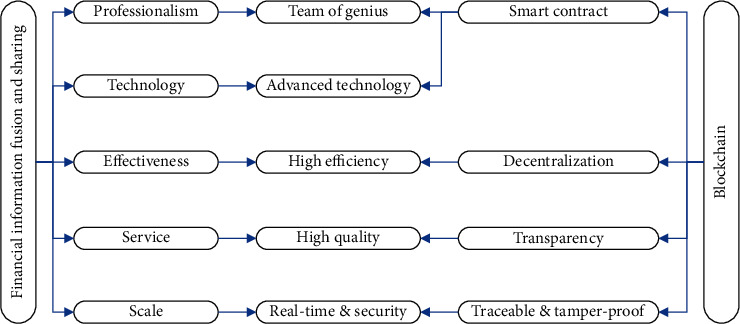
Technical architecture of system.

The reason why blockchain technology can be applied to financial information fusion and sharing systems is because of their compatibility. The high-efficiency requirements emphasized by the financial information fusion, and sharing system is precisely matched by the decentralization characteristics and automatic execution functions of blockchain technology. Because there is no step-by-step instruction in the block-two technology, each instruction can be executed at the same time. The openness and transparency of blockchain technology greatly reduces the time for business transactions, and the trust mechanism of blockchain technology perfectly fits with the trust required for financial sharing. The professionalism emphasized by the fusion and sharing of financial information requires a professional talent team and advanced science and technology as technical support. The emergence of smart contracts has subtly transformed the traditional financial model's reliance on manual labor into a reliance on intelligent data. The emergence of blockchain avoids the adverse impact on business processes due to trust issues and is used to standardize the relevant data for its financial sharing. The security and timeliness of financial shared data is guaranteed by the traceability and tamper-proof features of blockchain technology. At present, for enterprises, financial costs, management and control difficulties, and operational risks are constantly increasing. The traditional financial sharing model seems to have failed to meet the daily operation needs of enterprises, and it is urgent to continue to reform and innovate. The application of blockchain technology can break through the development bottleneck of sharing corporate financial information at this stage and optimize the internal processes of the company. This guarantees the accuracy and security of data, and improves the internal control level and work efficiency of the enterprise.  Figure 1 shows the technical architecture of the blockchain technology and financial information fusion and sharing system.

The enterprise financial information fusion and sharing system adopts a decentralized P2P network architecture. All enterprise branches that join the blockchain, through application, register as a node on the enterprise financial information fusion and sharing system. Under the blockchain technology, there is no limit to the number of nodes in the enterprise financial information fusion and sharing system, which means that the scope of enterprise services will be further expanded after the introduction of blockchain technology. The enterprise financial information fusion and sharing system optimizes the financial business processing process and is cost-effective. The unique decentralization and trustless characteristics of blockchain play a positive role in improving cost-effectiveness. The malleability of the blockchain is also due to the underlying infrastructure, so smart contracts can be implemented and set up using this technology. This can ensure the automatic execution of any business process in the financial operation of the enterprise and can avoid excessive reliance on manpower. It can be seen that the shared service platform and blockchain technology are not mutually exclusive. From this point of view, it is feasible to apply blockchain technology to the enterprise financial information fusion and sharing system.

### 3.3. Necessity of Blockchain Technology Application

The introduction of blockchain technology can avoid the bloated problem in the implementation of financial sharing, optimize the performance of the system from multiple aspects, and meet the needs of enterprise financial management in the new era. After applying blockchain technology to the enterprise financial information fusion and sharing system, the process and quality of financial sharing can be greatly optimized. First, after the financial sharing model is established, transaction information is stored in the form of business documents. Under the blockchain technology, the data of each node realize mutual verification, which greatly reduces the workload of auditors and avoids redundant audit work. Therefore, the introduction of blockchain not only helps to solve the complex auditing problems of financial sharing centers in the past but also fundamentally subverts the way of auditing business data. This transforms an after-the-fact audit into an after-the-fact audit, further streamlining the organization's processes. With the existence of the blockchain, transaction information flows directly from suppliers and customers to the enterprise, eliminating the need for intermediate links and improving the efficiency of financial management within the enterprise. Second, under the original financial sharing model, business documents record all business information left by the transaction management process. Under the blockchain technology, the problem of information asymmetry can be greatly alleviated. In addition, if a staff member wants to modify a certain information, it must be approved by all nodes of the blockchain before it can be executed. This mechanism fundamentally eliminates accounting fraud. At the same time, the data of each block on the main blockchain cover timestamps and are arranged in a chain structure, which ensures the traceability of business terminal information. Third, under the blockchain technology, the enterprise financial information fusion and sharing system becomes a huge distributed ledger. Financial information is backed up at each node. Even if the data of any node are accidently lost, other nodes will retain a complete chain of information. In addition, the public and private keys of the blockchain are responsible for data encryption and decryption, respectively, which is equivalent to data locking during data transmission. In order to obtain real financial data information, a real-time data receiver with a private key is required to ensure the authenticity and security of financial data.

### 3.4. Enterprise Financial Information Fusion and Sharing System

For enterprises, their financial information fusion and sharing system is mainly responsible for some basic work, and its practitioners are generally grassroots financial supervisors. The three-tier financial organization of the financial sharing service is optimized according to the characteristics of the blockchain. This work replaces the original financial sharing service platform with data blockchain and business blockchain. Among them, the data blockchain and business blockchain are mainly responsible for providing group financial data information for the strategic blockchain and providing business support. The strategic blockchain provides relevant information such as investment risk and decision prediction for the strategic decision making of the enterprise financial information fusion and sharing system, thereby helping it to better avoid risks and make decisions. After the information is reviewed and sorted by the strategic service center, it is used by the management.

**Figure 2:**
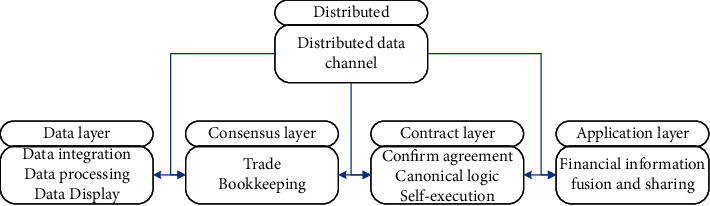
Specific architecture of system.

Financial sharing + the underlying structure of the blockchain can be obtained by transforming the infrastructure of the blockchain. This structure is the key to ensuring the rational use of blockchain technology in the enterprise financial information fusion and sharing system, and is also the key to optimizing blockchain financial sharing services. The actuality of the block financial sharing service can only be guaranteed if the underlying architecture is established. The specific architecture is shown in Figure 2.

The main content of the data layer includes the collection of records and detailed information of the internal current account payment of the enterprise. Recording here refers to recording all aspects of it, including transaction object number, transaction quantity, sending and receiving addresses, and transaction timestamp. Each block exists in the form of an end-to-end Hash function, which creates favorable conditions for data information to be untamperable and unforgeable, and completes the full traceability of the transaction process. In the entire blockchain structure, the data layer is the entire foundation, and it is responsible for data representation, data processing, and data integration in financial shared services. In the process of value or fund transfer, information such as timestamp, transaction address, transaction object, and other kinds of information together form a block, relying on key technologies such as encryption mechanism and hash function to ensure data security.

The PoW consensus mechanism in blockchain technology conducts keen competition through the distributed distribution of nodes, ensuring the unification of data and the reliability of consensus. In a shared service system, if you want to ensure the credibility and decentralization that the blockchain introduces with lag, a PoS consensus mechanism must be developed. For financial information sharing and development service enterprises, the PoS consensus mechanism can provide an efficient and convenient consensus mechanism, and can change its accounting and transaction authentication methods. This eliminates the previous cumbersome approval process and positions the financial center in financial sharing to build an environment of trust and achieve the goal of stable operation.

In the running state of the blockchain mode, all businesses are automatically executed according to the contract, and the entire contract layer is centered on the smart contract. The operation logic, execution conditions, processing procedures, and core elements of the core financial shared services are all included in the contract and automatically implemented in the business process.

The application layer refers to providing technical support for programming according to the actual work needs in the enterprise financial information fusion and sharing service, so as to better integrate the blockchain technology and the financial data sharing service platform and improve the financial service level of the enterprise.

### 3.5. Double-Chain Architecture in Enterprise Branch

**Figure 3:**
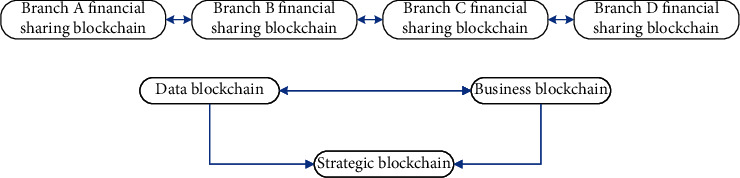
Double-chain architecture in enterprise branch.

The blockchain-based enterprise financial information fusion and sharing system is composed of three parts: data blockchain, business blockchain, and strategic blockchain, forming a unique dual-chain model between branches. As far as enterprise companies are concerned, each branch is used as each block on the intercompany blockchain to form a double-chain blockchain, as demonstrated in Figure 3.

Official departments exist as regulators and supervise specific business behaviors on the chain to prevent the occurrence of internal 51% attack risks. When some companies in the group join, members on the chain can communicate in advance and study and agree on their entry thresholds, thus ensuring the security of data on the chain. In the early stage of a new company joining, a voting session can be set up. When non-51% of the votes pass, all companies on the chain must reach an agreement before allowing the new company to obtain the qualification to enter the enterprise blockchain. In this way, the authenticity and accuracy of corporate financial data are guaranteed.

### 3.6. Benefit Analysis for System

In the process of analyzing the enterprise financial information fusion and sharing system, the AHP analytic hierarchy process is used in this paper. In addition, I wanted to do it and proposed four dimensions: information sharing dimension, group financial system dimension, enterprise organization process dimension, and other factor dimensions, and each dimension has its corresponding weight. Compare the four dimensions of the two models, then score each dimension, and finally multiply the obtained score by the weight to obtain the final conclusion.

The overall evaluation system is shown in Table 1. The customer dimension selects customer growth rate, financial service satisfaction, and risk control level as the three indicators of the program layer. The group financial dimension includes three indicators: group financial shared cost, labor cost, and financial service level. For the enterprise organization process dimension, it includes three aspects: business process optimization, business error rate, and employee business processing efficiency. The other dimensions select three indicators: employee turnover rate, customer satisfaction, and corporate training investment.

Under the analytic hierarchy process, under the principle of rationality of weight distribution, the allocation of performance evaluation indicators is completed, and a scientific evaluation system structure is formed. The operation of all enterprises is inseparable from the support of customers. Therefore, the customer dimension occupies the first place in the four dimensions of the criterion layer, the group financial dimension and the enterprise organization process dimension share the second place, and the final dimension is another factor. The ultimate purpose of an enterprise's business execution is to obtain economic benefits, and the application of blockchain technology to its financial sharing platform mainly optimizes its internal processes and reduces the internal costs of the enterprise. And all of this is for customer service, so the customer dimension is the most important. The control of corporate organizational processes and the group's financial aspects are also adjusted from within the group to provide customers with better services. Throughout the ages, customer resources have been regarded as the core and fundamental of all enterprise operations. Based on the above analysis and discussion, this work has formulated an enterprise index evaluation system to achieve long-term strategic planning.  Table 2 is the overall layer weight and judgment matrix. C is customer, GF is group finance, BOF is business organization process, and OF is another factor.

Then, we calculate the maximum characteristic root of the judgment matrix and then calculate the consistency index.

(1) $$\begin{array}{c} CI=\frac{\left(\lambda_{\max}-n\right)}{\left(n-1\right)}\text{,} \end{array}$$

where *λ*_max_ is maximum characteristic root.

The random consistency ratio is then calculated.

(2) $$\begin{array}{c} CR=\frac{CI}{RI}\text{,} \end{array}$$

where *RI* is the mean stochastic consistency index.

Finally, the weight of each indicator can be calculated.

### 3.7. Measures to Ensure the System

First, we pay attention to the trend of national policies in real time and ensure their implementation. For enterprise companies, national policies and regulations are the basis for establishing a financial information fusion and sharing system. Especially for large group companies, institutional guarantees and support are even more important. The professional technology and information technology advantages of enterprises have been advocated by the state, and the government is currently encouraging enterprises to establish their own financial sharing platforms, thereby accelerating the transformation of the accounting industry to intelligence. The development of blockchain technology is in full swing now, and finance plays an inestimable role for a group enterprise, so strict requirements are generally formulated. Today, the blockchain is not perfect, and its defects affect its integration with the financial sharing platform, so government departments need to strengthen support in this regard. For the management department, because the blockchain technology is relatively new, it should continue to strengthen its supervision. Or we formulate unified regulations, policies, and standard systems to ensure scientific and rationality. According to the characteristics of blockchain, it can be classified into the scope of traditional financial market rules; that is, it can be supervised by conventional regulatory means.

Second, we provide stable capital flow to ensure platform update and optimization. In fact, during the creation of a blockchain financial information fusion and sharing system, a large capital investment is generally required. Especially in the early stage of project operation, a large amount of capital flow is required to maintain the operation of its platform. Therefore, in the early stage of introducing the blockchain financial sharing platform system, enterprises should raise funds reasonably to ensure the stable development of their supporting systems. Under the environment of accelerated upgrading of information technology, financial sharing and upgrading relying on technological development will be more frequent in the future, and each upgrade will have different requirements for technology. This requires the continuous strengthening of vocational training of technical personnel, which is also a large expenditure for enterprises. The blockchain technology financial sharing platform is not yet mature in enterprise application, but in the period of strategic planning. Everything starts from scratch, and the establishment of the laboratory, the preparation of the infrastructure, and the training of professionals all require a certain amount of capital.

Third, we improve the training system to create professional talents. The lack of professional talents is the main problem faced during the construction of the blockchain financial information fusion and sharing system. The introduction of blockchain technology has put forward higher technical requirements for enterprise talents. The professional ability and professional quality of the traditional accounting talent team cannot meet the construction of the new financial sharing platform, which has a greater impact on the operating results of the financial sharing platform. This means that it is the general trend to actively cultivate accounting compound talents, so as to meet the needs of modern society for the registration of accountants, and during the upgrade and transformation of accounting work, we can also obtain talent guarantees. Technical talents are an important part of blockchain technology, and in the financial sharing platform, relevant practitioners should also put blockchain technology in an important position on the basis of understanding accounting expertise. Because under the blockchain technology, every step of financial sharing is inseparable from the optimization of the blockchain, so it requires the staff to master the blockchain technology needed in the actual work. At the same time, with the promotion of blockchain technology, the problem of unclear responsibilities of the corporate financial sharing platform will be solved. Driven by blockchain technology, data are open and transparent, and manual work is greatly reduced. On the one hand, it can reduce the boring and complicated work to mobilize the enthusiasm of employees. On the other hand, it can also simplify the work process and clarify the responsibilities. In this dimension, we must ensure the sustainable supply of professional talents and ensure the orderly operation of the platform.

## 4. Experiment

### 4.1. Performance Analysis on Designed System

This work evaluates the performance of the blockchain-based enterprise financial information fusion and sharing system. The main evaluation indicators are bandwidth overhead, transmission rate, maximum delay, minimum delay, average delay, and throughput.

**Figure 4:**
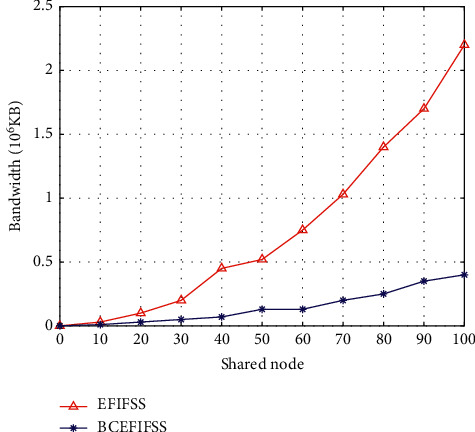
Bandwidth overhead comparison of EFIFSS and BCEFIFSS.

In this work, the enterprise financial information fusion and sharing system that does not use blockchain technology is named EFIFSS, and the system embedded in the blockchain is named BCEFIFSS. This work first compares the bandwidth overhead of EFIFSS and BCEFIFSS, and the comparison results are demonstrated in Figure 4.

**Figure 5:**
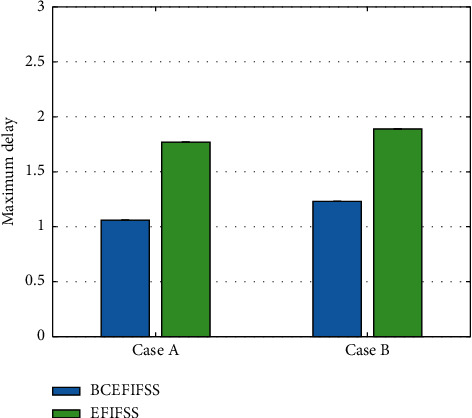
Maximum delay comparison of EFIFSS and BCEFIFSS.

As the number of shared nodes increases, the bandwidth of both systems increases. However, compared with the shared system without blockchain, the BCEFIFSS system using blockchain technology has smaller bandwidth consumption. After that, this work compares the maximum delay of the two systems, and the detailed data comparison is demonstrated in Figure 5.

**Figure 6:**
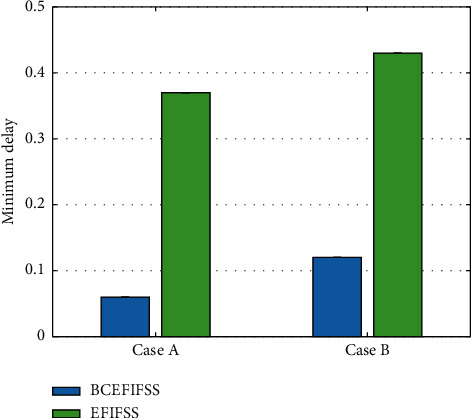
Minimum delay comparison of EFIFSS and BCEFIFSS.

After using the blockchain integration technology, the maximum delay of the system has dropped significantly, and the overall maximum delay has dropped by about 30%. Similar to the maximum delay, the minimum delay is also an important performance indicator. This work continues to compare the minimum delays of the two systems, as illustrated in Figure 6.

**Figure 7:**
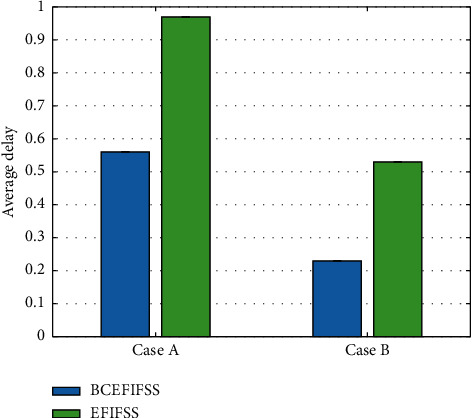
Average delay comparison of EFIFSS and BCEFIFSS.

After using the blockchain integration technology, the minimum delay of the system has dropped significantly, and the overall maximum delay has dropped by about 70% to 90%. The average delay is also another important performance indicator. This work continues to compare the average delay of the two systems, as demonstrated in Figure 7.

**Figure 8:**
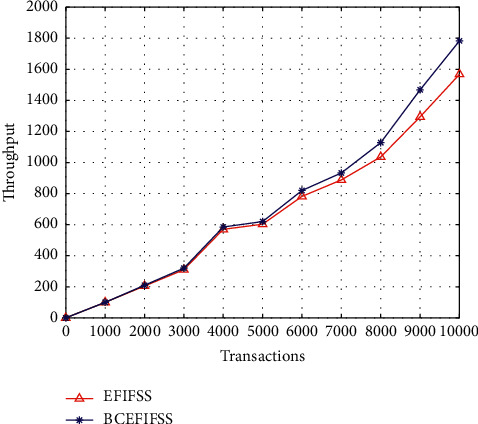
Throughput comparison of EFIFSS and BCEFIFSS.

After using the blockchain integration technology, the average delay of the system has dropped significantly, and the overall maximum delay has dropped by about 50%. Finally, this work compares the throughput of the two systems, which is an important performance indicator to measure a hardware and software system. The comparison data are demonstrated in Figure 8.

The throughput of both systems showed an upward trend as transaction volumes increased. But compared with systems that do not use blockchain, with blockchain technology embedded, the throughput is greater.

### 4.2. Analysis on Benefit

To analyze the benefits of the blockchain-based enterprise financial information fusion and sharing system, this work first uses AHP to determine the weights of indicators of different dimensions. The weights of the four dimensions of customer, group finance, enterprise organization process, and other factors are determined as shown in Table 3.

The customer dimension has the highest weight, followed by the enterprise organization process, followed by the group finance dimension, and the other factor dimensions have the lowest weight. The weight indicators of the customer dimension are demonstrated in Table 4.

The most important indicator is the customer growth rate indicator, which accounts for 0.561. This is due to the fact that under the blockchain technology, due to its decentralized characteristics, it can meet the real-time nature of information transmission. The weight indicators of the group finance dimension are demonstrated in Table 5.

For the construction of enterprise financial sharing platform, in the dimension of group finance, it mainly includes three aspects: financial sharing cost, labor cost, and financial service level. Compared with enterprises, the labor cost is far less than the benefits brought by the construction of the financial sharing center, and its benefits far exceed the cost of construction. The weight indicators of the enterprise organization process dimension are demonstrated in Table 6.

The organizational process of the enterprise is very important to the construction and operation of the financial sharing platform. In the dimension of enterprise organizational process, the indicator of business process optimization is the most important, and the efficiency of employee business processing is the least weighted. The weight indicators of the other factor dimension are demonstrated in Table 7.

The employee turnover rate of an enterprise is the most critical indicator among other factors, and the weights of the other two indicators are not much different and should be retained.

**Figure 9:**
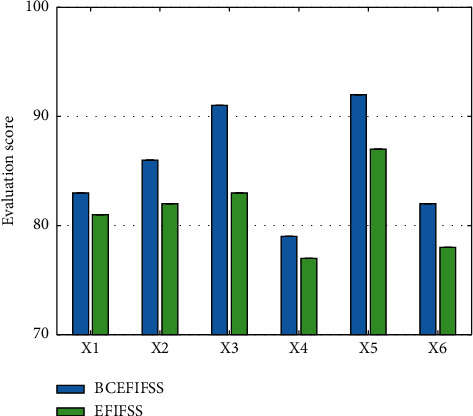
Comparison of the first six indicators.

**Figure 10:**
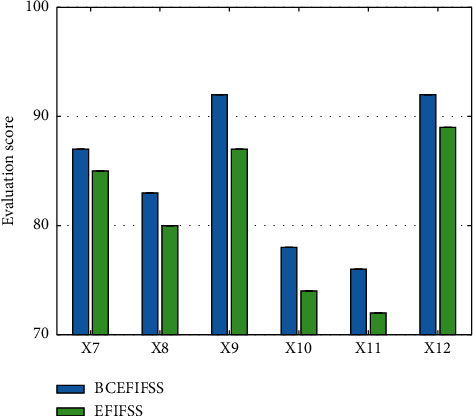
Comparison of the last six indicators.

After determining the weights of various indicators, this work compares the benefits of the shared system after using the blockchain and the traditional shared system. The comparison data are demonstrated in Figures 9 and 10.

In these 12 benefit analysis indicators, after the enterprise financial information fusion and sharing system using blockchain technology, the score of each indicator has increased, and the final overall score is 82.5 and 70.8, respectively. These data verify the feasibility and superiority of this work.

## 5. Conclusion

In the era of information technology, the credit crisis has become an important resistance point that plagues the current financial business. As a result, it provides a place for the blockchain that relies on its distributed encrypted account book, and the application of blockchain technology will definitely have a significant impact on the current accounting industry ecology and operating model. Accounting information, as an important symbol to record all the current accounts of an enterprise, is destined to become one of the important indicators of the ecological transformation of the accounting industry. Blockchain technology skillfully applies advanced technologies such as unified algorithms, encryption technology, and smart contracts to establish an anonymous credit mechanism and form a high-tech distributed ledger. In a huge distributed ledger, point-to-point targeted transaction methods and network technology can greatly improve the work efficiency of accountants. Encrypted storage technology greatly improves the security and transparency of financial analysis data, and significantly reduces the cost of financial information. At the same time, another unique feature of blockchain technology is that it has a unique timestamp function, which can promote enterprises to optimize their internal workflow, and then promote enterprises to move toward the road of information transformation. The fusion and sharing of financial information has become a necessary development strategy for centralizing and optimizing management, strengthening internal control systems, and improving business performance in the era of big data. This work first analyzes the necessity and feasibility of applying blockchain technology to the enterprise financial information fusion and sharing system. Then, it specifically describes how the blockchain technology is applied to the enterprise financial information fusion and sharing system, and improves the enterprise financial information fusion and sharing system through the blockchain technology. This forms an enterprise financial information fusion and sharing system between companies, which improves the limitations and disadvantages of the current financial sharing platform. Finally, using the AHP to analyze and compare the indicators of the traditional financial sharing model and the financial information fusion sharing model under the blockchain technology, it is concluded that the blockchain technology brings positive changes to the enterprise financial information fusion sharing model.

## Figures and Tables

**Table 1:** Overall evaluation system.

Criterion layer	Program
Customer	Customer growth rate
	Financial service satisfaction
	Risk control level

Group finance	Financial shared cost
	Labor cost
	Financial service level

Business organization process	Business process optimization
	Business error rate and
	Industrial business processing efficiency

Other factors	Turnover rate
	Customer satisfaction
	Corporate training investment

**Table 2:** Pairwise discriminant matrix.

Item	C	GF	BOP	OF
C	1.00	2.00	2.00	3.00
GF	0.50	1.00	2.00	2.00
BOP	0.50	0.50	1.00	2.00
OF	0.33	0.33	0.50	1.00

**Table 3:** Assignment of weights to criteria.

Index	Weight
Customer	0.42
Group finance	0.27
Enterprise organization process	0.19
Other factors	0.12

**Table 4:** Assignment of weights to customer dimension.

Index	Weight
Customer growth rate	0.61
Financial services satisfaction	0.29
Risk control level	0.10

**Table 5:** Assignment of weights to group finance dimension.

Index	Weight
Financial shared cost	0.62
Labor cost	0.22
Financial service level	0.12

**Table 6:** Assignment of weights to enterprise organization process dimension.

Index	Weight
Business process optimization	0.49
Business error rate and	0.36
Industrial business processing efficiency	0.15

**Table 7:** Assignment of weights to other factor dimensions.

Index	Weight
Turnover rate	0.46
Customer satisfaction	0.33
Corporate training investment	0.21

## Data Availability

The datasets used during the current study can be obtained from the author upon reasonable request.
